# Correlation Between Surrogate End Points and Overall Survival in a Multi-institutional Clinicogenomic Cohort of Patients With Non–Small Cell Lung or Colorectal Cancer

**DOI:** 10.1001/jamanetworkopen.2021.17547

**Published:** 2021-07-26

**Authors:** Kenneth L. Kehl, Gregory J. Riely, Eva M. Lepisto, Jessica A. Lavery, Jeremy L. Warner, Michele L. LeNoue-Newton, Shawn M. Sweeney, Julia E. Rudolph, Samantha Brown, Celeste Yu, Philippe L. Bedard, Deborah Schrag, Katherine S. Panageas

**Affiliations:** 1 Department of Medical Oncology, Division of Population Sciences, Dana-Farber Cancer Institute, Harvard Medical School, Boston, Massachusetts; 2 Department of Epidemiology and Biostatistics, Memorial Sloan Kettering Cancer Center, New York, New York; 3 Department of Medicine, Division of Hematology/Oncology, Vanderbilt-Ingram Cancer Center, Vanderbilt University Medical Center, Nashville, Tennessee; 4 Department of Biomedical Informatics, Vanderbilt-Ingram Cancer Center, Vanderbilt University Medical Center, Nashville, Tennessee; 5 Vanderbilt-Ingram Cancer Center, Vanderbilt University Medical Center, Nashville, Tennessee; 6 American Association for Cancer Research, Philadelphia, Pennsylvania; 7 Division of Medical Oncology & Hematology, Princess Margaret Cancer Centre/University Health Network, Toronto, Ontario, Canada; 8 Department of Medicine, University of Toronto, Toronto, Ontario, Canada; 9 Associate Editor, *JAMA*

## Abstract

**Importance:**

Contemporary observational cancer research requires associating genomic biomarkers with reproducible end points; overall survival (OS) is a key end point, but interpretation can be challenging when multiple lines of therapy and prolonged survival are common. Progression-free survival (PFS), time to treatment discontinuation (TTD), and time to next treatment (TTNT) are alternative end points, but their utility as surrogates for OS in real-world clinicogenomic data sets has not been well characterized.

**Objective:**

To measure correlations between candidate surrogate end points and OS in a multi-institutional clinicogenomic data set.

**Design, Setting, and Participants:**

A retrospective cohort study was conducted of patients with non–small cell lung cancer (NSCLC) or colorectal cancer (CRC) whose tumors were genotyped at 4 academic centers from January 1, 2014, to December 31, 2017, and who initiated systemic therapy for advanced disease. Patients were followed up through August 31, 2020 (NSCLC), and October 31, 2020 (CRC). Statistical analyses were conducted on January 5, 2021.

**Exposures:**

Candidate surrogate end points included TTD; TTNT; PFS based on imaging reports only; PFS based on medical oncologist ascertainment only; PFS based on either imaging or medical oncologist ascertainment, whichever came first; and PFS defined by a requirement that both imaging and medical oncologist ascertainment have indicated progression.

**Main Outcomes and Measures:**

The primary outcome was the correlation between candidate surrogate end points and OS.

**Results:**

There were 1161 patients with NSCLC (648 women [55.8%]; mean [SD] age, 63 [11] years) and 1150 with CRC (647 men [56.3%]; mean [SD] age, 54 [12] years) identified for analysis. Progression-free survival based on both imaging and medical oncologist documentation was most correlated with OS (NSCLC: ρ = 0.76; 95% CI, 0.73-0.79; CRC: ρ = 0.73; 95% CI, 0.69-0.75). Time to treatment discontinuation was least associated with OS (NSCLC: ρ = 0.45; 95% CI, 0.40-0.50; CRC: ρ = 0.13; 95% CI, 0.06-0.19). Time to next treatment was modestly associated with OS (NSCLC: ρ = 0.60; 0.55-0.64; CRC: ρ = 0.39; 95% CI, 0.32-0.46).

**Conclusions and Relevance:**

This cohort study suggests that PFS based on both a radiologist and a treating oncologist determining that a progression event has occurred was the surrogate end point most highly correlated with OS for analysis of observational clinicogenomic data.

## Key Points

**Question:**

What surrogate end point for capturing worsening disease is most correlated with overall survival (OS) in large linked clinicogenomic data sets?

**Findings:**

In this cohort study of patients with non–small cell lung cancer or colorectal cancer who initiated systemic therapy for advanced disease, progression-free survival based on both radiologist and medical oncologist assessment was more consistently correlated with OS than other candidate end points, including time to treatment discontinuation and time to next treatment.

**Meaning:**

This study suggests that, based on its correlation with OS, progression-free survival based on both radiologist and medical oncologist assessment may be an optimal surrogate end point for analysis of observational clinicogenomic data in cancer research.

## Teaser

This cohort study measures associations between candidate surrogate end points and overall survival in a multi-institutional clinicogenomic data set of patients with non–small cell lung cancer (NSCLC) or colorectal cancer.

## Introduction

For patients with cancer enrolled in clinical trials, the Response Evaluation Criteria in Solid Tumors, version 1.1,^1^ are applied to ascertain treatment response and disease progression. However, outside of clinical trials, clinical outcomes are typically recorded only in the unstructured text of radiology reports and clinician progress notes.^2^ This can present challenges to the reproducibility of cancer research that incorporates large quantities of molecular and genomic data now routinely generated across institutions.^3^

Although overall survival (OS) constitutes a key outcome in cancer research, end points such as progression-free survival (PFS; time to progression or death), time to treatment discontinuation (TTD), and time to next treatment (TTNT) may also be relevant in some contexts. Progression can be assessed earlier than mortality, and PFS is less associated with subsequent lines of therapy, which can confound ascertainment of the association of any individual treatment with OS. However, the process of extracting PFS outcomes from electronic health record (EHR) data at scale is resource intensive, requiring review of thousands of clinical documents. Measurement of PFS in observational contexts is also not standardized regarding the definition of cancer progression. In contrast, TTD, defined as the time from initiation of a systemic therapy regimen to the date of treatment discontinuation or death, can be rapidly extracted from structured pharmacy data and has therefore been proposed as an alternative end point in the observational setting.^4,5^ Time to next treatment, defined as time from initiation of a systemic therapy to the date of initiation of the first subsequent systemic therapy regimen^6,7,8^ or death, can similarly be extracted from pharmacy data.

The utility of end points such as PFS, TTD, or TTNT, particularly when they are applied in lieu of, or as a surrogate for, OS, may be evaluated by measuring the correlation between the end point and OS.^9^ For example, disease-free survival has become an accepted surrogate end point in adjuvant therapy clinical trials for colorectal cancer (CRC) owing to its high correlation with OS.^10,11^ Understanding the correlation between PFS measures and OS, and other pragmatic end points (TTD or TTNT) and OS, specifically in the observational context may have increasing implications given the growing role of real-world evidence for regulatory purposes.^12^ A structured framework is necessary to define progression outcomes using such real-world data. The PRISSMM (pathology, radiology and imaging, documentation of signs and symptoms, medical oncologist notes, and tumor markers) framework consists of directives for abstracting clinical outcomes from individual components of the medical record.^13^ This system fosters transparency and reproducibility by defining outcome measures with specific attention to data provenance. To specifically inform research derived from linked clinical and genomic data, this study focused on a multi-institutional cohort of patients with advanced non–small cell lung cancer (NSCLC) or CRC whose tumor sequencing results were submitted to the American Association for Cancer Research’s Project GENIE (Genomics Evidence Neoplasia Information Exchange).^3^ The specific objective of this analysis was to report correlations between OS and TTD, TTNT, and PFS, as systematically ascertained from the EHR using the PRISSMM framework for medical record curation.

## Methods

### Cohort

The cohort for this analysis included patients with stage I to stage IV NSCLC or CRC whose tumors underwent genomic sequencing at Dana-Farber Cancer Institute, Memorial Sloan Kettering Cancer Center, Princess Margaret Cancer Centre, or the Vanderbilt-Ingram Cancer Center between January 1, 2014, and December 31, 2017. Patients consented to medical record review and genomic profiling of their tumor tissue at each institution; this supplemental retrospective analysis was approved by the Dana-Farber/Harvard Cancer Center institutional review board under a waiver of informed consent because this study presented no more than minimal risk to the participants. Outcomes were analyzed for patients who were either diagnosed initially with stage IV NSCLC or CRC and received at least 1 systemic therapy regimen, or who initially received a diagnosis of stage I to stage III NSCLC or CRC and received at least 1 systemic therapy regimen that began at least 6 months after initial diagnosis (assumed to represent treatment for recurrent disease). Patients were followed up through August 31, 2020 (NSCLC), and October 31, 2020 (CRC). This study is reported according to the Strengthening the Reporting of Observational Studies in Epidemiology (STROBE) reporting guideline.^14^

### Medical Record Curation

Curation of imaging reports and medical oncologist notes was performed according to the PRISSMM framework.^13^ For each imaging report and medical oncologist assessment, curators were asked to record whether the radiologist or clinician described the presence of cancer, and if so, whether the cancer was improving and responding, stable, mixed, or worsening or progressing. Curators reviewed the text of radiologists’ reports for computed tomography, magnetic resonance imaging, positron emission tomography, and nuclear medicine evaluations. Curators reviewed the first note per month from a medical oncologist; if one was not available, a note from an advanced practice clinician (nurse practitioner or physician assistant) was reviewed.

### Outcomes

#### Time to Treatment Discontinuation

The index date for TTD was defined as the start date of the first systemic therapy regimen, consisting of a drug or group of drugs for which there was documentation from the treating oncologist of a plan for simultaneous administration for recurrent or metastatic disease. Dates of initiation and final administration of each drug in infusional regimens were curated. For NSCLC, the most common first-line infusional regimens were given every 3 weeks, so to more closely capture the point when a decision was reached to discontinue the regimen, the end date of infusional regimens was defined for this analysis as 3 weeks after the last drug in the regimen was administered.^15^ For CRC, the most common first-line infusional regimens were given every 2 weeks, so the end date for infusional regimens was defined as 2 weeks after the last drug in the regimen was administered. For oral therapy, the end date was defined as the date on which the prescription expired or the medical oncologist documented discontinuation of the regimen, whichever came first. In primary analyses, death also constituted a treatment discontinuation event. Censoring was performed on the date patients were last known alive and receiving treatment.

#### Time to Next Treatment

In primary analyses, TTNT was defined as time from first-line treatment start to initiation of subsequent systemic therapy or death. Censoring was performed at the date patients were last known alive and free of subsequent therapy.

#### Progression-Free Survival

Four definitions of PRISSMM-derived PFS outcomes were evaluated: PFS-imaging (PFS-I; time to first worsening or progression documented in imaging report, or death), PFS–medical oncologist (PFS-M; time to first worsening or progression documented in medical oncologist assessment, or death), PFS-I-or-M (time to first indication of worsening or progression in imaging report or medical oncologist assessment, or death, whichever was earliest), and PFS-I-and-M (time from treatment start to worsening or progression having been documented in both an imaging report and a medical oncologist assessment, or death). The index date for PFS was defined as the start date of first-line therapy for recurrent or metastatic disease. Patients were censored on the date last known alive and free of disease progression.

### Statistical Analysis

#### End Point Correlations

Analyses were conducted on January 5, 2021. Overall survival, TTD, TTNT, and PFS measures were estimated using the Kaplan-Meier method. Correlations and 95% CIs between OS and (1) TTD, (2) TTNT, or (3) each PRISSMM-derived PFS outcome were measured using normal scores rank correlation, calculated using the iterative multiple imputation approach for analysis of correlations between 2 partially censored failure times.^16^ Among patients with NSCLC, correlations between OS and alternative end points were further explored by systemic therapy regimen category, including (1) all regimens, (2) cytotoxic chemotherapy only (with or without an anti–vascular endothelial growth factor agent), (3) checkpoint inhibitor immunotherapy only, or (4) oral targeted therapy. Among patients with CRC, only the analysis of all regimens was performed, because most regimens in that cohort were chemotherapy based.

In sensitivity analyses, correlations were recalculated after restricting to patients who underwent genomic testing prior to starting first-line therapy. Because all patients in this cohort had genomic testing as an inclusion criterion, this procedure restricted calculations to the time during which patients were at risk for death, and it was performed in lieu of left truncation, which the statistical package used for correlation calculations could not incorporate. In a second set of sensitivity analyses, patients who died without experiencing disease progression or a treatment discontinuation or change event were excluded from the denominator to assess the extent to which correlations between candidate outcomes and OS were owing to mortality rather than progression or treatment discontinuation events. Calculations were performed using R, version 3.6.1 (R Group for Statistical Computing) and the SurvCorr R package, version 1.0.^17^ All *P* values were from 2-sided tests and results were deemed statistically significant at *P* < .05.

## Results

### Cohorts

There were 1161 patients with NSCLC (648 women [55.8%]; mean [SD] age, 63 [11] years) and 1150 patients with CRC (647 men [56.3%]; mean [SD] age, 54 [12] years) (eFigures 1 and 2 in Supplement 1). Additional patient characteristics are provided in Table 1.

**Table 1.** Cohort Characteristics

Characteristic	Patients, No. (%)	
	NSCLC (n = 1161)	CRC (n = 1150)
Histologic characteristics		
NSCLC		
Nonsquamous	1009 (86.9)	NA
Squamous	98 (8.4)	NA
Not otherwise specified	54 (4.7)	NA
Molecular alterations		
Alteration		
* KRAS*	316 (27.2)	518 (45.0)
* NRAS*	NA	62 (5.4)
* EGFR*	306 (26.4)	NA
* BRAF*	57 (4.9)	116 (10.1)
* MET*	56 (4.8)	NA
Rearrangement		
* ALK*	43 (3.7)	NA
* ROS1*	21 (1.8)	NA
* RET*	13 (1.1)	NA
None of the above	482 (34.6)	482 (41.9)
Sex		
Male	513 (44.2)	647 (56.3)
Female	648 (55.8)	503 (43.7)
Age, y		
<40	32 (2.8)	143 (12.4)
40-49	104 (9.0)	274 (23.8)
50-59	293 (25.2)	340 (29.6)
60-69	401 (34.5)	281 (24.4)
70-79	282 (24.3)	88 (7.7)
≥80	49 (4.2)	24 (2.1)
Stage at diagnosis		
I-III	404 (34.8)	461 (40.1)
IV	757 (65.2)	689 (59.9)
Treating institution		
Dana-Farber Cancer Institute	409 (35.2)	390 (33.9)
Memorial Sloan Kettering Cancer Center	552 (47.5)	558 (48.5)
Princess Margaret Cancer Centre	41 (3.5)	NA
Vanderbilt University Medical Center	159 (13.7)	202 (17.6)

Abbreviations: CRC, colorectal cancer; NA, not applicable; NSCLC, non–small cell lung cancer.

### Patient-Level End Point Correlations After First-Line Therapy

#### Non–Small Cell Lung Cancer

The median OS after initiation of any first-line therapy was 28.9 months (interquartile range [IQR], 12.0-66.6 months) (Table 2); in a sensitivity analysis restricted to 375 patients starting therapy after their genomic testing report, median OS was 22.8 months (IQR, 9.5-55.4 months) (eTable 1 in Supplement 1). Among all 1161 patients, TTD yielded the shortest median time to event, at 3.6 months (IQR, 1.6-8.5 months), while PFS-I-and-M yielded the longest median time to event at 9.6 months (IQR, 4.5-20.8 months) (Table 2).

**Table 2.** Correlations Between Clinical End Points and OS by Therapy Regimen Category

Characteristic	Time to event, median (IQR), mo	ρ (95% CI)
**NSCLC: all regimens**		
No.	1161	NA
OS	28.9 (12.0-66.6)	1 [Reference]
TTD	3.6 (1.6-8.5)	0.45 (0.40-0.50)
TTNT	7.1 (2.8-16.2)	0.60 (0.55-0.64)
PFS-I	6.5 (2.5-15.1)	0.63 (0.59-0.67)
PFS-M	8.4 (3.8-18.2)	0.71 (0.67-0.74)
PFS-I-or-M	5.6 (1.9-12.3)	0.61 (0.56-0.65)
PFS-I-and-M	9.6 (4.5-20.8)	0.76 (0.73-0.79)
**CRC: all regimens**		
No.	1150	NA
OS	42.0 (22.8-83.8)	1 [Reference]
TTD	4.3 (2.3-6.5)	0.13 (0.06-0.19)
TTNT	7.2 (3.9-6.5)	0.39 (0.32-0.46)
PFS-I	10.5 (5.6-19.1)	0.66 (0.61-0.70)
PFS-M	13.5 (7.7-26.3)	0.71 (0.67-0.74)
PFS-I-or-M	10.0 (5.1-17.4)	0.65 (0.61-0.69)
PFS-I-and-M	14.4 (8.6-28.9)	0.73 (0.69-0.75)
**NSCLC: cytotoxic chemotherapy only**		
No.	683	NA
OS	27.3 (11.0-66.5)	1 [Reference]
TTD	3.0 (1.9-5.5)	0.34 (0.26-0.41)
TTNT	6.1 (2.4-13.4)	0.53 (0.46-0.60)
PFS-I	6.9 (2.8-15.4)	0.66 (0.61-0.70)
PFS-M	8.1 (4.0-17.1)	0.70 (0.66-0.74)
PFS-I-or-M	5.9 (2.3-12.3)	0.63 (0.57-0.67)
PFS-I-and-M	9.3 (4.6-21.0)	0.76 (0.72-0.79)
**NSCLC: immunotherapy only**		
No.	124	NA
OS	18.6 (7.1-43.9)	1 [Reference]
TTD	4.1 (1.6-8.9)	0.76 (0.64-0.84)
TTNT	5.2 (2.1-12.8)	0.70 (0.57-0.80)
PFS-I	2.6 (1.4-10.2)	0.63 (0.46-0.75)
PFS-M	2.0 (1.4-13.7)	0.69 (0.52-0.80)
PFS-I-or-M	1.9 (0.9-6.8)	0.59 (0.43-0.72)
PFS-I-and-M	4.5 (1.7-14.5)	0.78 (0.65-0.86)
**NSCLC: oral targeted therapy**		
No.	207	NA
OS	47.0 (22.8-91.4)	1 [Reference]
TTD	12.0 (7.4-26.0)	0.63 (0.50-0.72)
TTNT	12.6 (7.6-26.9)	0.62 (0.51-0.71)
PFS-I	9.7 (5.1-19.6)	0.52 (0.38-0.64)
PFS-M	12.7 (7.1-27.4)	0.63 (0.51-0.73)
PFS-I-or-M	8.5 (4.7-19.1)	0.50 (0.36-0.62)
PFS-I-and-M	14.4 (7.7-30.8)	0.71 (0.59-0.80)

Abbreviations: CRC, colorectal cancer; IQR, interquartile range; NA, not applicable; NSCLC, non–small cell lung cancer; OS, overall survival; PFS-I, PFS based on imaging reports only; PFS-I-and-M, PFS defined by a requirement that both imaging and medical oncologist ascertainment have indicated progression; PFS-I-or-M, PFS based on either imaging or medical oncologist ascertainment, whichever came first; PFS-M, PFS based on medical oncologist ascertainment only; TTD, time to treatment discontinuation (or death); TTNT, time to next treatment (or death).

**Figure 1.**
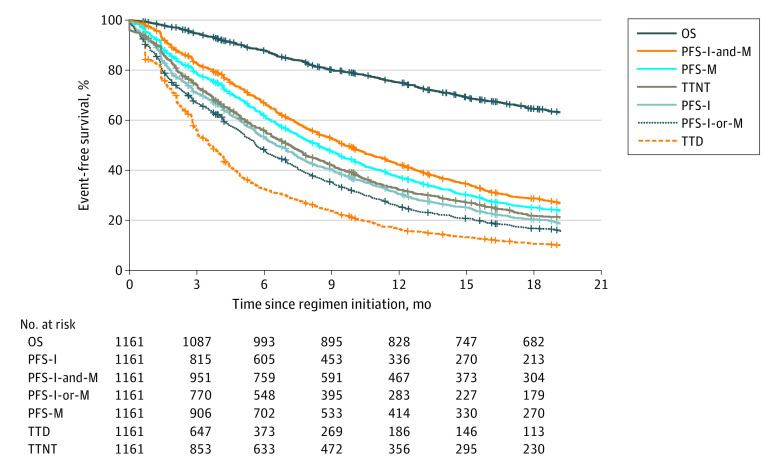
Kaplan-Meier Curves for Candidate Outcome Measures After First-Line Systemic Therapy for Recurrent or Metastatic Non–Small Cell Lung Cancer OS indicates overall survival; PFS, progression-free survival; PFS-I, PFS based on imaging reports only; PFS-I-and-M, PFS defined by a requirement that both imaging and medical oncologist ascertainment have indicated progression; PFS-I-or-M, PFS based on either imaging or medical oncologist ascertainment, whichever came first; PFS-M, PFS based on medical oncologist ascertainment only; TTD, time to treatment discontinuation (or death); and TTNT, time to next treatment (or death).

Time to treatment discontinuation had the weakest correlation with OS (ρ = 0.45; 95% CI, 0.40-0.50) and PFS-I-and-M had the strongest correlation (ρ = 0.76; 95% CI, 0.73-0.79) (Table 2). Time to next treatment was modestly associated with OS (ρ = 0.60; 0.55-0.64). A similar pattern held when the analysis was restricted to the 375 patients initiating therapy after genomic testing; eTable 1 in Supplement 1. Kaplan-Meier curves for OS, TTD, TTNT, and each variant of PFS are provided in Figure 1. The correlation between the 2 individual PFS measures themselves (PFS-I and PFS-M) was 0.69 (95% CI, 0.65-0.72).

The most common treatment regimens administered in this cohort are listed in eTable 2 in Supplement 1. Among 683 patients whose first-line therapy was cytotoxic chemotherapy only, TTD was poorly correlated with OS (ρ = 0.34; 95% CI, 0.26-0.41), as was TTNT (ρ = 0.53; 95% CI, 0.46-0.60) (Table 2). However, these end points were more correlated with OS among 124 patients who began first-line immunotherapy only (TTD: ρ = 0.76; 95% CI, 0.64-0.84; TTNT: ρ = 0.70; 95% CI, 0.57-0.80). Among patients receiving cytotoxic chemotherapy only and those receiving immunotherapy only, PFS-I-and-M was similarly correlated with OS (chemotherapy: ρ = 0.76; 95% CI, 0.72-0.79; immunotherapy: ρ = 0.78; 95% CI, 0.65-0.86).

In a second sensitivity analysis in which patients who died before experiencing another outcome event were excluded from correlation calculations, death in the absence of progression within this cohort was uncommon, even for PFS-I-and-M, which by definition has the longest time to event among the candidate PFS measures (ie, 23 of 1161 patients [2.0%]) and for TTNT, which could be impacted by high rates of death without receiving a subsequent line of therapy (25 of 1161 patients [2.2%]). Correlation coefficients were similar to the primary analysis (eTable 3 in Supplement 1).

#### Colorectal Cancer

The median OS after initiation of any first-line therapy for patients with CRC was 42.0 months (IQR, 22.8-83.8 months) (Table 2); in a sensitivity analysis restricted to 160 patients starting therapy after their genomic testing report to remove time when patients were not at risk for death, it was not reached (IQR, 18.2 months to not reached) (eTable 1 in Supplement 1). Among all 1150 patients, TTD yielded the shortest median time to event, at 4.3 months (IQR, 2.3-6.5 months), while PFS-I-and-M yielded the longest median time to event at 14.4 months (IQR, 8.6-28.9 months) (Table 2).

**Figure 2.**
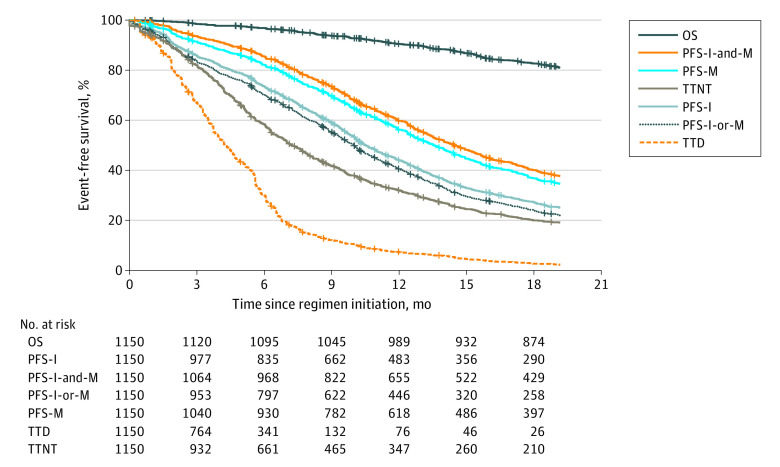
Kaplan-Meier Curves for Candidate Outcome Measures After First-Line Systemic Therapy for Recurrent or Metastatic Colorectal Cancer OS indicates overall survival; PFS, progression-free survival; PFS-I, PFS based on imaging reports only; PFS-I-and-M, PFS defined by a requirement that both imaging and medical oncologist ascertainment have indicated progression; PFS-I-or-M, PFS based on either imaging or medical oncologist ascertainment, whichever came first; PFS-M, PFS based on medical oncologist ascertainment only; TTD, time to treatment discontinuation (or death); and TTNT, time to next treatment (or death).

Patterns of correlation between end points for CRC were similar to those in the NSCLC cohort. For CRC, TTD had the weakest correlation with OS (ρ = 0.13; 95% CI, 0.06-0.19) and PFS-I-and-M had the strongest correlation (ρ = 0.73; 95% CI, 0.69-0.75) (Table 2). Time to next treatment was modestly associated with OS (ρ = 0.39; 95% CI, 0.32-0.46). A similar pattern held when the analysis was restricted to the 160 patients initiating therapy after genomic testing (eTable 1 in Supplement 1). Kaplan-Meier curves for OS, TTD, TTNT, and each variant of PFS are provided in Figure 2. The correlation between the 2 individual PFS measures (PFS-I and PFS-M) was 0.73 (95% CI, 0.70-0.75).

In a second sensitivity analysis in which patients who died before experiencing another outcome event were excluded from correlation calculations, death in the absence of progression remained uncommon, even for PFS-I-and-M (ie, 8 of 1150 patients [0.7%]) and for TTNT (7 of 1150 patients [0.6%]). Correlation coefficients were similar to the primary analysis (eTable 3 in Supplement 1).

## Discussion

In this multi-institutional analysis of candidate clinical end points among patients undergoing systemic therapy for advanced, genomically characterized NSCLC or CRC, TTD was poorly correlated with OS and TTNT was modestly correlated with OS. Progression-free survival estimated based on abstraction of both imaging and medical oncologist notes (PFS-I-and-M) was most consistently correlated with OS. The magnitude of these patient-level correlations between alternative end points and OS was similar to that observed in analyses of clinical trial data^4^ and an observational cohort^8,18^ of patients with NSCLC, although the examination of outcomes derived from specific components of the health record across cancer types was a novel feature of the present study. Surrogate outcomes may be useful in observational data sets even when OS data are available, particularly when researchers study a specific line of therapy in contexts in which survival through multiple lines of therapy is common. The consistency of the correlation between PFS-I-and-M and OS in this analysis implies that when a surrogate outcome in clinicogenomic analyses for patients with NSCLC or CRC is required, PFS-I-and-M may be an optimal way to define real-world PFS.

Although this analysis focused on a clinicogenomic data set, it has implications for analysis of real-world evidence in observational cancer research in general. Time to treatment discontnuation and TTNT are attractive end points because they can often be measured with minimal manual review of medical records using structured pharmacy records. In contrast, PFS-I and PFS-M require review of radiology reports and oncologist notes to identify evidence of progression. These results suggest that, in some contexts, a consistent framework for abstraction of such clinical end points, with attention to data provenance^13^—whether progression is documented on imaging results, by a clinician, or both—may be required for observational cancer research, rather than relying on TTD or TTNT. This is a substantial challenge because the careful manual abstraction of EHR data is very resource intensive. One solution is to develop validated natural language processing methods for extracting these end points from the EHR^19^; the feasibility of such approaches for both imaging reports^20,21^ and clinician notes^22,23^ has been previously demonstrated.

Correlations between surrogate end points and OS likely varied by treatment modality owing to several clinical factors. For example, planned discontinuation of therapy after a given number of cycles, prior to clinical progression, could partially account for a poor correlation between TTD and OS among patients receiving chemotherapy. An extreme example of this dynamic would include therapy delivered once, without immediate plans for regular administration of the drug, as might occur when floxuridine is used as local therapy for liver metastases in patients with CRC.^24^ In addition, discontinuation of treatment for toxic effects may further diminish the correlation between TTD and OS if toxic effects are less associated with mortality risk than is progressive disease. This dynamic may explain the modest correlation between TTNT and OS as well because treatment discontinued for toxic effects may be followed by initiation of other treatment prior to progression. On the other hand, for patients with NSCLC, TTD and OS may have been correlated for patients receiving immunotherapy in this cohort if single-agent checkpoint inhibitor treatment had a low rate of severe adverse events^18,25^ and therefore less early discontinuation. In this analysis, the patient-level correlation between TTD and OS among patients receiving oral targeted therapy for NSCLC was higher than that among patients receiving chemotherapy, again potentially owing to lower severe adverse event rates with targeted therapy,^26,27^ but PFS-I-and-M remained numerically most correlated with OS after oral targeted therapy as well.

### Strengths and Limitations

This study has some strengths, including its multi-institutional cohort of patients with 2 types of common solid tumors who had linked clinical and genomic data. In such data sets, thorough evaluation of clinical end points is particularly critical, because the data are rich enough to enable researchers to ask a wide variety of questions about the association between genomic markers, treatment exposures, and outcomes.^13^

This study also has some limitations, including forms of selection bias. Contributing institutions were 4 North American academic centers, which may not be representative of institutions where patients receive care in the community or in other parts of the world. These patients were also selected for tumor genomic profiling, such that, particularly for NSCLC, those whose tumors may be less likely to harbor targetable mutations may have been underrepresented.

Patients do not always receive all their care within 1 institution or health system. Data on outcome events ascertained outside the primary academic centers for this analysis could potentially have been less complete than data for internally ascertained outcomes. In addition, patients who are included in a clinicogenomic cohort, by definition, very rarely have tumor genomic profiling performed after death. This can introduce lead time between diagnosis and genomic sequencing during which patients included in the data set could not have died, inflating survival estimates. Applying methods for handling left truncation—entering patients into the risk set of a time-to-event analysis only after they have undergone genomic profiling^28^—is a solution to this problem, but measuring correlations between left-truncated time-to-event outcomes requires the development of novel statistical methods. This is especially a challenge if tumor genotyping is performed selectively at the point of clinical progression, which can result in informative left truncation, or temporal selection bias, in which patients selectively enter the cohort specifically because they are at increasing risk of a poor outcome.^29^ Nevertheless, these results were similar in sensitivity analyses that were restricted to patients receiving treatments initiated after genomic testing, such that all patients were in the risk set at the index time point. In addition, in observational data sets, the frequency of clinical end point ascertainment is based on the judgment of a patient’s clinician and may itself be associated with clinical risk over time; the association of this phenomenon with outcome estimates requires further study.

Despite the relatively large size of the cohorts analyzed in this study, there were overlapping 95% CIs for some comparisons, particularly between PFS-I-and-M and PFS-M for both NSCLC and CRC, and by category of systemic therapy, in which each category had a more limited sample size. An even larger study would be needed to formally compare the correlations between these 2 particular end points and OS and evaluate the generalizability of all observed correlations to additional cancer types and categories of treatment, because the validity of surrogate end points may be specific to disease contexts^30^ and clinical questions.^31^ More broadly, substantial literature exists on approaches to evaluating the validity of surrogate end points in the context of clinical trials,^31,32^ focused largely on measuring correlations between treatment effects. Many observational research questions, particularly in the clinicogenomic context, are focused less on treatment effect comparisons than on biomarker associations. More robust frameworks for evaluating surrogate end points in the observational context^33^ are needed.

## Conclusions

In this observational cohort of patients with genomically profiled advanced NSCLC or CRC, TTD was inconsistently correlated, and TTNT moderately correlated, with OS on a per-patient basis. Progression-free survival based on review of both imaging reports and medical oncologist notes was most correlated with OS among patients with either type of cancer. Although TTD and TTNT are straightforward to calculate owing to the availability of structured pharmacy data in the EHR, these results indicate that researchers should be cautious about applying such end points if a surrogate outcome consistently correlated with OS is required. In such contexts, PFS-I-and-M may be an optimal choice.

## Notes

Supplement 1. **eTable 1.** Correlations Between Surrogate Outcomes and Overall Survival by Therapy Regimen Category: Sensitivity Analysis Restricted to Patients Who Started First-Line Therapy After Genomic Testing **eTable 2.** Most Common Systemic Therapy Regimens Administered as First-Line Therapy **eTable 3.** Correlations Between Surrogate Outcomes and Overall Survival by Therapy Regimen Category: Sensitivity Analysis Excluding Patients Who Died Without Experiencing a Surrogate Outcome Event **eFigure 1.** Cohort Derivation: Non–Small Cell Lung Cancer (NSCLC) **eFigure 2.** Cohort Derivation: Colorectal Cancer (CRC)

Supplement 2. **Nonauthor Collaborators.** AACR Project GENIE Consortium Members
