# Supplementary material for: Correlation Between Surrogate End Points and Overall Survival in a Multi-institutional Clinicogenomic Cohort of Patients With Non–Small Cell Lung or Colorectal Cancer
Source: JAMA Netw Open. 2021 Jul 26;4(7):e2117547. doi: 10.1001/jamanetworkopen.2021.17547 (PMC8314138; doi:10.1001/jamanetworkopen.2021.17547)
Supplement: Supplement 1. — eTable 1. Correlations Between Surrogate Outcomes and Overall Survival by Therapy Regimen Category: Sensitivity Analysis Restricted to Patients Who Started First-Line Therapy After Genomic Testing eTable 2. Most Common Systemic Therapy Regimens Administered as First-Line Therapy eTable 3. Correlations Between Surrogate Outcomes and Overall Survival by Therapy Regimen Category: Sensitivity Analysis Excluding Patients Who Died Without Experiencing a Surrogate Outcome Event eFigure 1. Cohort Derivation: Non–Small Cell Lung Cancer (NSCLC) eFigure 2. Cohort Derivation: Colorectal Cancer (CRC) [file jamanetwopen-e2117547-s001.pdf]

Supplementary Online Content

Kehl KL, Riely GJ, Lepisto EM, et al; American Association of Cancer Research (AACR) Project Genomics Evidence Neoplasia Information Exchange (GENIE) Consortium. Association between surrogate end points and overall survival in a multi-institutional clinicogenomic cohort of patients with non–small cell lung or colorectal cancer. *JAMA Netw Open.* 2021;4(7):e2117547. doi:10.1001/jamanetworkopen.2021.17547

eTable 1. Correlations Between Surrogate Outcomes and Overall Survival by Therapy Regimen Category: Sensitivity Analysis Restricted to Patients Who Started First-Line Therapy After Genomic Testing
eTable 2. Most Common Systemic Therapy Regimens Administered as First-Line Therapy
eTable 3. Correlations Between Surrogate Outcomes and Overall Survival by Therapy Regimen Category: Sensitivity Analysis Excluding Patients Who Died Without Experiencing a Surrogate Outcome Event
eFigure 1. Cohort Derivation: Non–Small Cell Lung Cancer (NSCLC)
eFigure 2. Cohort Derivation: Colorectal Cancer (CRC)

This supplementary material has been provided by the authors to give readers additional information about their work.

© 2021 Kehl KL et al. *JAMA Network Open.*

eTable 1. Correlations Between Surrogate Outcomes and Overall Survival by Therapy Regimen Category: Sensitivity Analysis Restricted to Patients Who Started First-Line Therapy After Genomic Testing

	Index date: Start of first-line therapy		
	N	Median time to event, months (IQR)	Rho (correlation with OS) (95% CI)
NSCLC: All regimens			
OS	375	22.8 (9.5-55.4)	1.0
TTD		3.3 (1.4-8.7)	0.53 (0.44-0.61)
TTNT		6.7 (2.3-17.5)	0.66 (0.57-0.73)
PFS-I		5.6 (1.9-15.1)	0.68 (0.60-0.74)
PFS-M		6.9 (2.9-21.0)	0.69 (0.62-0.75)
PFS-I-or-M		4.9 (1.4-12.3)	0.62 (0.54-0.69)
PFS-I-and-M		8.1 (3.7-22.9)	0.79 (0.73-0.84)
CRC: All regimens			
OS	160	NR (18.2-NR)	1.0
TTD		4.4 (2.0-6.6)	0.29 (0.08-0.47)
TTNT		11.4 (3.7-NR)	0.64 (0.44-0.78)
PFS-I		10.0 (3.7-47.6)	0.77 (0.64-0.86)
PFS-M		13.9 (7.2-49.1)	0.79 (0.66-0.87)
PFS-I-or-M		10.0 (3.2-31.1)	0.75 (0.61-0.85)
PFS-I-and-M		14.3 (7.5-49.1)	0.82 (0.71-0.90)
NSCLC: Cytotoxic chemotherapy only			
OS	170	20.3 (6.6-53.2)	1.0
TTD		2.8 (1.7-4.2)	0.40 (0.25-0.53)
TTNT		4.9 (2.0-13.0)	0.57 (0.40-0.70)
PFS-I		5.6 (2.5-14.7)	0.73 (0.63-0.80)
PFS-M		6.0 (3.0-16.8)	0.69 (0.59-0.77)
PFS-I-or-M		4.5 (1.9-11.1)	0.66 (0.56-0.75)
PFS-I-and-M		6.6 (3.7-18.4)	0.77 (0.68-0.84)
NSCLC: Immunotherapy only			
OS	66	20.8 (7.2-43.9)	1.0
TTD		4.0 (2.1-10.9)	0.72 (0.52-0.84)

© 2021 Kehl KL et al. *JAMA Network Open.*

TTNT		6.3 (2.5-25.1)	0.71 (0.51-0.84)
PFS-I		3.2 (1.4-13.8)	0.66 (0.44-0.80)
PFS-M		4.2 (1.4-13.8)	0.65 (0.44-0.79)
PFS-I-or-M		1.9 (1.2-11.5)	0.58 (0.31-0.76)
PFS-I-and-M		5.3 (1.7-15.8)	0.76 (0.57-0.87)
NSCLC: Oral targeted therapy			
OS	73	37.2 (18.5-NR)	1.0
TTD		10.8 (6.3-38.7)	0.67 (0.44-0.81)
TTNT		11.9 (7.0-28.2)	0.70 (0.52-0.82)
PFS-I		8.1 (5.0-22.9)	0.59 (0.34-0.77)
PFS-M		16.8 (6.8-36.7)	0.74 (0.42-0.90)
PFS-I-or-M		7.8 (4.9-22.9)	0.57 (0.30-0.76)
PFS-I-and-M		16.8 (7.4-36.7)	0.83 (0.69-0.91)

Legend to Supplemental Table 1: NSCLC, non-small cell lung cancer; CRC, colorectal cancer; OS, overall survival; TTD, time to treatment discontinuation (or death); TTNT, time to next treatment, or death; PFS-I, time to progression annotated on an imaging report, or death; PFS-M, time to progression annotated on a medical oncologist note, or death; PFS-I-or-M, time to progression annotated on either an imaging report or medical oncologist note, or death; PFS-I-and-M, time to progression having been annotated on both an imaging report and a medical oncologist note, or death; NR, not reached.

© 2021 Kehl KL et al. *JAMA Network Open.*

eTable 2. Most Common Systemic Therapy Regimens Administered as First-Line Therapy

Non-small cell lung cancer (N=1161)		Colorectal cancer (N=1150)	
Regimen	N	Regimen	N
Carboplatin + pemetrexed	210	FOLFOX	408
Erlotinib	116	FOLFOX + bevacizumab	228
Carboplatin + paclitaxel	83	FOLFIRI + bevacizumab	129
Carboplatin + pemetrexed + pembrolizumab	80	FOLFIRI	76
Cisplatin + pemetrexed	72	Capecitabine	55
Pembrolizumab	70	FOLFOXIRI	28
Single-agent clinical trial regimen	52	Capecitabine + oxaliplatin	27
Dual-agent clinical trial regimen	43	Floxuridine	27
Nivolumab	42	5-FU + leucovorin	19
Cisplatin + pemetrexed + bevacizumab	34	Capecitabine + oxaliplatin + bevacizumab	13
Crizotinib	31	5-FU	13
Carboplatin + gemcitabine	25	Irinotecan + bevacizumab	10
Cisplatin + etoposide	23		
Osimertinib	21		
Carboplatin + pemetrexed + pembrolizumab	20		
Other	239	Other	117

© 2021 Kehl KL et al. *JAMA Network Open.*

eTable 3. Correlations Between Surrogate Outcomes and Overall Survival by Therapy Regimen Category: Sensitivity Analysis Excluding Patients Who Died Without Experiencing a Surrogate Outcome Event

	N (%)	Median time to event, months (IQR)	Rho (correlation with OS) (95% CI)
NSCLC: All regimens			
OS	1161	28.9 (12.0-66.6)	1.0
TTD	1145	3.7 (1.6-8.6)	0.41 (0.36-0.47)
TTNT	1136	7.2 (2.9-16.6)	0.59 (0.53-0.64)
PFS-I	1152	6.5 (2.5-15.2)	0.64 (0.59-0.68)
PFS-M	1139	8.5 (4.1-18.4)	0.70 (0.66-0.74)
PFS-I-or-M	1153	5.7 (1.9-12.3)	0.61 (0.57-0.65)
PFS-I-and-M	1138	9.9 (4.7-21.2)	0.75 (0.72-0.79)
CRC: All regimens			
OS	1150	42.0 (22.8-83.8)	1.0
TTD	1146	4.3 (2.3-6.5)	0.12 (0.05-0.19)
TTNT	1143	7.2 (3.9-14.7)	0.39 (0.32-0.46)
PFS-I	1147	10.5 (5.7-19.1)	0.66 (0.61-0.70)
PFS-M	1142	13.5 (7.7-26.5)	0.71 (0.66-0.75)
PFS-I-or-M	1147	10.0 (5.1-17.4)	0.65 (0.60-0.70)
PFS-I-and-M	1142	14.4 (8.6-29.1)	0.73 (0.69-0.76)
NSCLC: Cytotoxic chemotherapy only			
OS	683	27.3 (11.0-66.5)	1.0
TTD	678	3.0 (1.9-5.6)	0.33 (0.26-0.40)
TTNT	669	6.1 (2.4-13.6)	0.53 (0.46-0.59)
PFS-I	677	6.9 (2.8-15.4)	0.66 (0.61-0.70)
PFS-M	669	8.4 (4.2-17.5)	0.70 (0.65-0.74)
PFS-I-or-M	677	5.9 (2.3-12.3)	0.63 (0.57-0.68)
PFS-I-and-M	669	9.3 (4.7-22.3)	0.75 (0.71-0.79)
NSCLC: Immunotherapy only			
OS	124	18.6 (7.1-43.9)	1.0

© 2021 Kehl KL et al. *JAMA Network Open.*

TTD	116	4.4 (2.1-9.0)	0.69 (0.49-0.81)
TTNT	118	5.2 (2.1-12.8)	0.68 (0.53-0.78)
PFS-I	122	2.5 (1.4-10.2)	0.63 (0.47-0.74)
PFS-M	121	4.1 (1.4-13.7)	0.68 (0.52-0.79)
PFS-I-or-M	123	1.9 (0.9-6.2)	0.56 (0.41-0.68)
PFS-I-and-M	120	4.5 (1.7-14.5)	0.77 (0.66-0.85)
NSCLC: Oral targeted therapy			
OS	207	47.0 (22.8-91.4)	1.0
TTD	207	12.0 (7.4-26.0)	0.64 (0.53-0.73)
TTNT	205	12.6 (7.7-26.9)	0.59 (0.47-0.69)
PFS-I	207	9.7 (5.1-19.6)	0.51 (0.35-0.65)
PFS-M	204	12.7 (7.1-27.3)	0.62 (0.44-0.76)
PFS-I-or-M	204	8.5 (4.7-19.1)	0.53 (0.40-0.64)
PFS-I-and-M	207	14.7 (7.8-30.6)	0.67 (0.52-0.78)

Legend to Supplemental Table 3: NSCLC, non-small cell lung cancer; CRC, colorectal cancer; OS, overall survival; TTD, time to treatment discontinuation (or death); TTNT, time to next treatment, or death; PFS-I, time to progression annotated on an imaging report, or death; PFS-M, time to progression annotated on a medical oncologist note, or death; PFS-I-or-M, time to progression annotated on either an imaging report or medical oncologist note, or death; PFS-I-and-M, time to progression having been annotated on both an imaging report and a medical oncologist note, or death.

© 2021 Kehl KL et al. *JAMA Network Open.*

eFigure 1. Cohort Derivation: Non–Small Cell Lung Cancer (NSCLC)

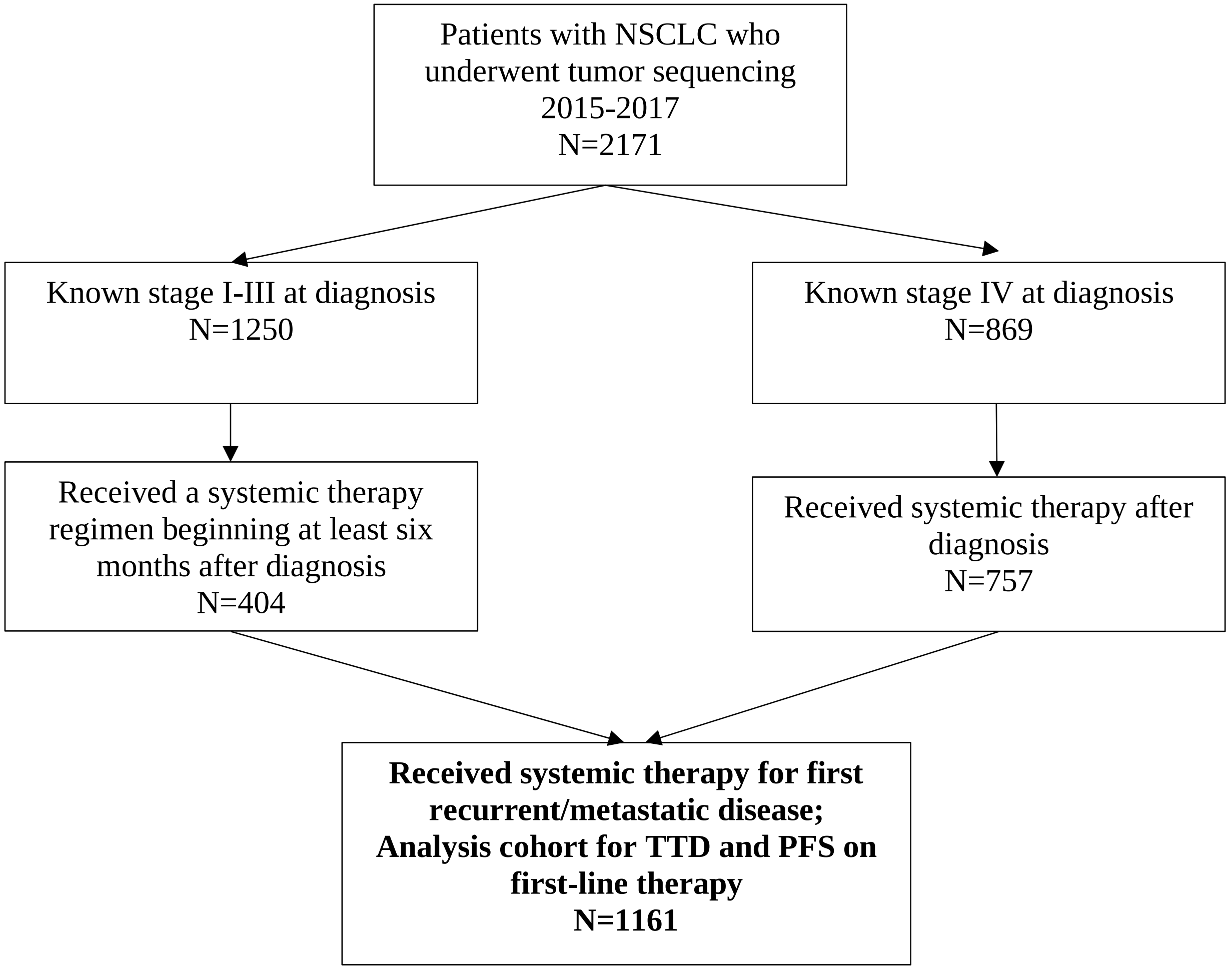

© 2021 Kehl KL et al. *JAMA Network Open.*

eFigure 2. Cohort Derivation: Colorectal Cancer (CRC)

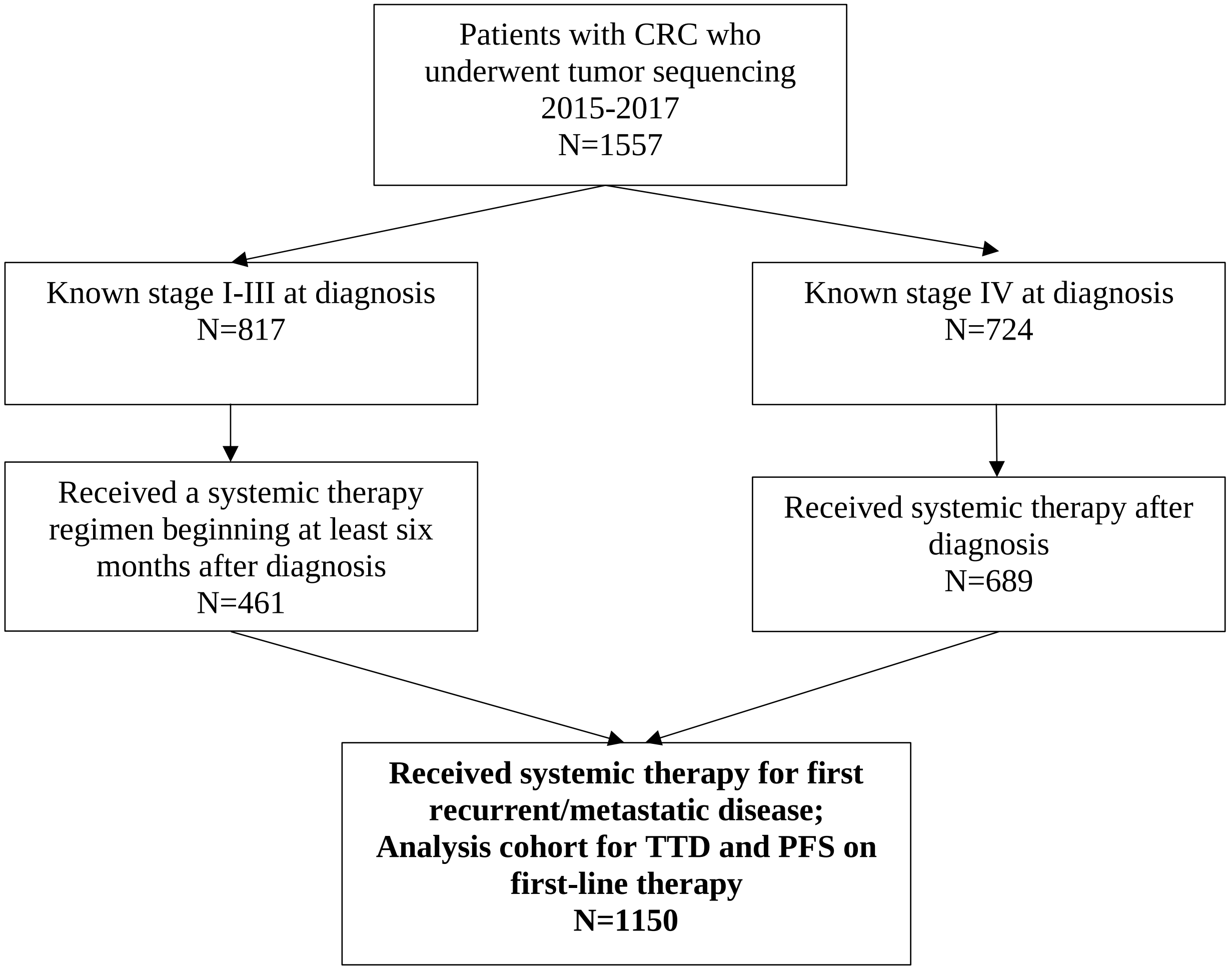

© 2021 Kehl KL et al. *JAMA Network Open.*
