# Supplementary material for: Correlation Between Surrogate End Points and Overall Survival in a Multi-institutional Clinicogenomic Cohort of Patients With Non–Small Cell Lung or Colorectal Cancer
Source: JAMA Netw Open. 2021 Jul 26;4(7):e2117547. doi: 10.1001/jamanetworkopen.2021.17547 (PMC8314138; doi:10.1001/jamanetworkopen.2021.17547)
Supplement: Supplement 2. — Nonauthor Collaborators. AACR Project GENIE Consortium Members [file jamanetwopen-e2117547-s002.pdf]

Supplemental Online Content: Nonauthor Collaborators

*Indicates required information. Only first name, last name, and suffix will appear in PubMed.

*Group Name: AACR Project GENIE Consortium							
***First Name and Middle Initial(s)**	***Last Name**	***Suffix (eg, Jr, III)**	Academic Degrees	Institution	Location (city, state/province, country)	Role or Contribution, eg, chair, principal investigator	Group (if more than 1 Group listed in the byline) and/or Subgroup (eg, Steering Committee)
Shawn	Sweeney			American Association for Cancer Research	Philadelphia, PA		
Margaret	Foti			American Association for Cancer Research	Philadelphia, PA		
Yekaterina	Khotskaya			American Association for Cancer Research	Philadelphia, PA		
Michael	Fiandalo			American Association for Cancer Research	Philadelphia, PA		
Benjamin	Gross			Memorial Sloan Kettering Cancer Center	New York, NY		
Nikolaus	Schultz			Memorial Sloan Kettering Cancer Center	New York, NY		
Brooke	Mastrogiacomo			Memorial Sloan Kettering Cancer Center	New York, NY		
Mahdi	Sarmardy			Children's Hospital of Philadelphia Cancer Center	Philadelphia, PA		
Marilyn	Li			Children's Hospital of Philadelphia Cancer Center	Philadelphia, PA		
Adam	Resnick			Children's Hospital of Philadelphia Cancer Center	Philadelphia, PA		
Angela	Waanders			Children's Hospital of Philadelphia Cancer Center	Philadelphia, PA		
Jena	Lilly			Children's Hospital of Philadelphia Cancer Center	Philadelphia, PA		
Richard	Carvajal			COLUMBIA	New York, NY		
Raul	Rabadan			COLUMBIA	New York, NY		
Matthew	Ingham			COLUMBIA	New York, NY		
Susan	Hsaio			COLUMBIA	New York, NY		

© 2020 American Medical Association. All rights reserved.

Supplemental Online Content: Nonauthor Collaborators

*Indicates required information. Only first name, last name, and suffix will appear in PubMed.

*First Name and Middle Initial(s)	*Last Name	*Suffix (eg, Jr, III)	Academic Degrees	Institution	Location (city, state/province, country)	Role or Contribution, eg, chair, principal investigator	Group (if more than 1 Group listed in the byline) and/or Subgroup (eg, Steering Committee)
Jean	Abraham			Cancer Research UK Cambridge Centre	Cambridge, UK		
James	Brenton			Cancer Research UK Cambridge Centre	Cambridge, UK		
Oscar	Rueda			Cancer Research UK Cambridge Centre	Cambridge, UK		
Carlos	Caldas			Cancer Research UK Cambridge Centre	Cambridge, UK		
Mikel	Valgañón			Cancer Research UK Cambridge Centre	Cambridge, UK		
Dilrini	Silva			Cancer Research UK Cambridge Centre	Cambridge, UK		
Chris	Boursnell			Cancer Research UK Cambridge Centre	Cambridge, UK		
Raquel	Garcia			Cancer Research UK Cambridge Centre	Cambridge, UK		
Ezequiel	Rodriguez			Cancer Research UK Cambridge Centre	Cambridge, UK		
Birgit	Nimmervoll			Cancer Research UK Cambridge Centre	Cambridge, UK		
Ethan	Cerami			Dana-Farber Cancer Institute	Boston, MA		
Matthew	Ducar			Dana-Farber Cancer Institute	Boston, MA		
Priti	Kumari			Dana-Farber Cancer Institute	Boston, MA		
Neal	Lindeman			Dana-Farber Cancer Institute	Boston, MA		
Laura	MacConnaill			Dana-Farber Cancer Institute	Boston, MA		
John	Orechia			Dana-Farber Cancer Institute	Boston, MA		
Deborah	Schrag			Dana-Farber Cancer Institute	Boston, MA		
Priyanka	Shivdasani			Dana-Farber Cancer Institute	Boston, MA		
Eliezer	Van Allen			Dana-Farber Cancer Institute	Boston, MA		
Jason	Johnson			Dana-Farber Cancer Institute	Boston, MA		

© 2020 American Medical Association. All rights reserved.

Supplemental Online Content: Nonauthor Collaborators

*Indicates required information. Only first name, last name, and suffix will appear in PubMed.

***First Name and Middle Initial(s)**	***Last Name**	***Suffix (eg, Jr, III)**	Academic Degrees	Institution	Location (city, state/province, country)	Role or Contribution, eg, chair, principal investigator	Group (if more than 1 Group listed in the byline) and/or Subgroup (eg, Steering Committee)
Pasi	Jänne			Dana-Farber Cancer Institute	Boston, MA		
Eva	Lepisto			Dana-Farber Cancer Institute	Boston, MA		
Michael	Hassett			Dana-Farber Cancer Institute	Boston, MA		
Sindy	Pimentel			Dana-Farber Cancer Institute	Boston, MA		
Parin	Sripakdeevong			Dana-Farber Cancer Institute	Boston, MA		
Katherine	Janeway			Dana-Farber Cancer Institute	Boston, MA		
Jason M.	Johnson			Dana-Farber Cancer Institute	Boston, MA		
Matthew	Meyerson			Dana-Farber Cancer Institute	Boston, MA		
Daniel	Quinn			Dana-Farber Cancer Institute	Boston, MA		
Oya	Cushing			Dana-Farber Cancer Institute	Boston, MA		
Kevin	Haigis			Dana-Farber Cancer Institute	Boston, MA		
Diana	Miller			Dana-Farber Cancer Institute	Boston, MA		
Kenneth	Kehl			Dana-Farber Cancer Institute	Boston, MA		
Alexander	Gustav			Dana-Farber Cancer Institute	Boston, MA		
Angela	Tramontano			Dana-Farber Cancer Institute	Boston, MA		
Simon Arango	Baquero			Dana-Farber Cancer Institute	Boston, MA		
Jonathan	Bell			Duke University Cancer Institute	Durham, NC		
Michelle	Green			Duke University Cancer Institute	Durham, NC		
Shannon	McCall			Duke University Cancer Institute	Durham, NC		
Michael	Datto			Duke University Cancer Institute	Durham, NC		
Fabien	Calvo			Gustave Roussy Cancer Campus	Villejuif, France		
Fabrice	Andre			Gustave Roussy Cancer Campus	Villejuif, France		
Meurice	Guillaume			Gustave Roussy Cancer Campus	Villejuif, France		

© 2020 American Medical Association. All rights reserved.

Supplemental Online Content: Nonauthor Collaborators

*Indicates required information. Only first name, last name, and suffix will appear in PubMed.

***First Name and Middle Initial(s)**	***Last Name**	***Suffix (eg, Jr, III)**	Academic Degrees	Institution	Location (city, state/province, country)	Role or Contribution, eg, chair, principal investigator	Group (if more than 1 Group listed in the byline) and/or Subgroup (eg, Steering Committee)
Semih	Dogan			Gustave Roussy Cancer Campus	Villejuif, France		
Lacroix	Ludovic			Gustave Roussy Cancer Campus	Villejuif, France		
Jean	Scoazec			Gustave Roussy Cancer Campus	Villejuif, France		
Monica	Ardenos			Gustave Roussy Cancer Campus	Villejuif, France		
Gilles	Vassal			Gustave Roussy Cancer Campus	Villejuif, France		
Stefan	Michels			Gustave Roussy Cancer Campus	Villejuif, France		
Victor	Velculescu			Sidney Kimmel Comprehensive Cancer Center at Johns Hopkins University	Baltimore, MD		
Alexander	Baras			Sidney Kimmel Comprehensive Cancer Center at Johns Hopkins University	Baltimore, MD		
Christopher	Gocke			Sidney Kimmel Comprehensive Cancer Center at Johns Hopkins University	Baltimore, MD		
Julie	Brahmer			Sidney Kimmel Comprehensive Cancer Center at Johns Hopkins University	Baltimore, MD		
Charles	Sawyers			Memorial Sloan Kettering Cancer Center	New York, NY		
David	Solit			Memorial Sloan Kettering Cancer Center	New York, NY		
Stu	Gardos			Memorial Sloan Kettering Cancer Center	New York, NY		

© 2020 American Medical Association. All rights reserved.

*Indicates required information. Only first name, last name, and suffix will appear in PubMed.

***First Name and Middle Initial(s)**	***Last Name**	***Suffix (eg, Jr, III)**	Academic Degrees	Institution	Location (city, state/province, country)	Role or Contribution, eg, chair, principal investigator	Group (if more than 1 Group listed in the byline) and/or Subgroup (eg, Steering Committee)
Mike	Berger			Memorial Sloan Kettering Cancer Center	New York, NY		
Marc	Ladanyi			Memorial Sloan Kettering Cancer Center	New York, NY		
Gregory	Riely			Memorial Sloan Kettering Cancer Center	New York, NY		
Joseph	Sirintrapun			Memorial Sloan Kettering Cancer Center	New York, NY		
Ari	Caroline			Memorial Sloan Kettering Cancer Center	New York, NY		
Joseph	Sirintrapun			Memorial Sloan Kettering Cancer Center	New York, NY		
Stacy	Thomas			Memorial Sloan Kettering Cancer Center	New York, NY		
Andrew	Zarski			Memorial Sloan Kettering Cancer Center	New York, NY		
Ahmet	Zehir			Memorial Sloan Kettering Cancer Center	New York, NY		
Alexia	Iasonosa			Memorial Sloan Kettering Cancer Center	New York, NY		
John	Philip			Memorial Sloan Kettering Cancer Center	New York, NY		
Samantha	Brown			Memorial Sloan Kettering Cancer Center	New York, NY		
Andrew	Kung			Memorial Sloan Kettering Cancer Center	New York, NY		
Ritika	Kundra			Memorial Sloan Kettering Cancer Center	New York, NY		
Julia	Rudolph			Memorial Sloan Kettering Cancer Center	New York, NY		

© 2020 American Medical Association. All rights reserved.

Supplemental Online Content: Nonauthor Collaborators

*Indicates required information. Only first name, last name, and suffix will appear in PubMed.

***First Name and Middle Initial(s)**	***Last Name**	***Suffix (eg, Jr, III)**	Academic Degrees	Institution	Location (city, state/province, country)	Role or Contribution, eg, chair, principal investigator	Group (if more than 1 Group listed in the byline) and/or Subgroup (eg, Steering Committee)
Jessica	Lavery			Memorial Sloan Kettering Cancer Center	New York, NY		
Hira	Rivzi			Memorial Sloan Kettering Cancer Center	New York, NY		
Julian	Schwartz			Memorial Sloan Kettering Cancer Center	New York, NY		
Caroline	McCarthy			Memorial Sloan Kettering Cancer Center	New York, NY		
Maufur	Bhuiya			Memorial Sloan Kettering Cancer Center	New York, NY		
Axel	Martin			Memorial Sloan Kettering Cancer Center	New York, NY		
Cynthia	Chu			Memorial Sloan Kettering Cancer Center	New York, NY		
Raymond	DuBois			Medical University of South Carolina	Charleston, SC		
Tony	van de Velde			The Netherlands Cancer Institute, Amsterdam, on behalf of the Center for Personalized Cancer Treatment	Utrecht, The Netherlands		
Geritt	Meijer			The Netherlands Cancer Institute, Amsterdam, on behalf of the Center for Personalized Cancer Treatment	Utrecht, The Netherlands		
Hugo	Horlings			The Netherlands Cancer Institute, Amsterdam, on behalf of the Center for Personalized Cancer Treatment	Utrecht, The Netherlands		

© 2020 American Medical Association. All rights reserved.

Supplemental Online Content: Nonauthor Collaborators

*Indicates required information. Only first name, last name, and suffix will appear in PubMed.

***First Name and Middle Initial(s)**	***Last Name**	***Suffix (eg, Jr, III)**	Academic Degrees	Institution	Location (city, state/province, country)	Role or Contribution, eg, chair, principal investigator	Group (if more than 1 Group listed in the byline) and/or Subgroup (eg, Steering Committee)
Harm	van Tinteren			The Netherlands Cancer Institute, Amsterdam, on behalf of the Center for Personalized Cancer Treatment	Utrecht, The Netherlands		
Martijn	Lolkema			The Netherlands Cancer Institute, Amsterdam, on behalf of the Center for Personalized Cancer Treatment	Utrecht, The Netherlands		
Les	Nijman			The Netherlands Cancer Institute, Amsterdam, on behalf of the Center for Personalized Cancer Treatment	Utrecht, The Netherlands		
Mariska	Bierkens			The Netherlands Cancer Institute, Amsterdam, on behalf of the Center for Personalized Cancer Treatment	Utrecht, The Netherlands		
Jelle	Hoeve			The Netherlands Cancer Institute, Amsterdam, on behalf of the Center for Personalized Cancer Treatment	Utrecht, The Netherlands		
Emilie	Voest			The Netherlands Cancer Institute, Amsterdam, on behalf of the Center for Personalized Cancer Treatment	Utrecht, The Netherlands		
Annemieke	Hiemstra			The Netherlands Cancer Institute, Amsterdam, on behalf of the Center for Personalized Cancer Treatment	Utrecht, The Netherlands		

© 2020 American Medical Association. All rights reserved.

Supplemental Online Content: Nonauthor Collaborators

*Indicates required information. Only first name, last name, and suffix will appear in PubMed.

***First Name and Middle Initial(s)**	***Last Name**	***Suffix (eg, Jr, III)**	Academic Degrees	Institution	Location (city, state/province, country)	Role or Contribution, eg, chair, principal investigator	Group (if more than 1 Group listed in the byline) and/or Subgroup (eg, Steering Committee)
Gabe	Sonke			The Netherlands Cancer Institute, Amsterdam, on behalf of the Center for Personalized Cancer Treatment	Utrecht, The Netherlands		
Jacques	Craenmehr			The Netherlands Cancer Institute, Amsterdam, on behalf of the Center for Personalized Cancer Treatment	Utrecht, The Netherlands		
Jan	Hudecek			The Netherlands Cancer Institute, Amsterdam, on behalf of the Center for Personalized Cancer Treatment	Utrecht, The Netherlands		
Kim	Monkhorst			The Netherlands Cancer Institute, Amsterdam, on behalf of the Center for Personalized Cancer Treatment	Utrecht, The Netherlands		
Walter	Urba			Providence Cancer Institute	Portland, Oregan		
Brady	Bernard			Providence Cancer Institute	Portland, Oregan		
Brian	Piening			Providence Cancer Institute	Portland, Oregan		
Carlo	Bifulco			Providence Cancer Institute	Portland, Oregan		
Paul	Tittel			Providence Cancer Institute	Portland, Oregan		
Julie	Cramer			Providence Cancer Institute	Portland, Oregan		
Justin	Guinney			SAGE Bionetworks	Seattle, WA		
Thomas	Yu			SAGE Bionetworks	Seattle, WA		
Xindi	Guo			SAGE Bionetworks	Seattle, WA		
Alyssa	Acebedo			SAGE Bionetworks	Seattle, WA		
Philip	Gold			Swedish Cancer Institute	Seattle, WA		
Neil	Bailey			Swedish Cancer Institute	Seattle, WA		
Sabah	Kadri			The University of Chicago Comprehensive Cancer Center	Chicago, IL		

© 2020 American Medical Association. All rights reserved.

*Indicates required information. Only first name, last name, and suffix will appear in PubMed.

***First Name and Middle Initial(s)**	***Last Name**	***Suffix (eg, Jr, III)**	Academic Degrees	Institution	Location (city, state/province, country)	Role or Contribution, eg, chair, principal investigator	Group (if more than 1 Group listed in the byline) and/or Subgroup (eg, Steering Committee)
Jeremy	Segal			The University of Chicago Comprehensive Cancer Center	Chicago, IL		
Wanjari	Pankhuri			The University of Chicago Comprehensive Cancer Center	Chicago, IL		
Peng	Wang			The University of Chicago Comprehensive Cancer Center	Chicago, IL		
Steinhardt	George			The University of Chicago Comprehensive Cancer Center	Chicago, IL		
Moung	Christine			University of California-San Francisco Helen Diller Family Comprehensive Cancer Center	San Francisco, CA		
Laura	Van't Veer			University of California-San Francisco Helen Diller Family Comprehensive Cancer Center	San Francisco, CA		
Eric	Talevich			University of California-San Francisco Helen Diller Family Comprehensive Cancer Center	San Francisco, CA		
Amanda	Wren			University of California-San Francisco Helen Diller Family Comprehensive Cancer Center	San Francisco, CA		
Alejandro	Sweet-Cordero			University of California-San Francisco Helen Diller Family Comprehensive Cancer Center	San Francisco, CA		
Michelle	Turski			University of California-San Francisco Helen Diller Family Comprehensive Cancer Center	San Francisco, CA		
Philippe	Bedard			Princess Margaret Cancer Centre	Toronto, Ontario, Canada		
Suzanne	KamelReid			Princess Margaret Cancer Centre	Toronto, Ontario, Canada		

© 2020 American Medical Association. All rights reserved.

Supplemental Online Content: Nonauthor Collaborators

*Indicates required information. Only first name, last name, and suffix will appear in PubMed.

***First Name and Middle Initial(s)**	***Last Name**	***Suffix (eg, Jr, III)**	Academic Degrees	Institution	Location (city, state/province, country)	Role or Contribution, eg, chair, principal investigator	Group (if more than 1 Group listed in the byline) and/or Subgroup (eg, Steering Committee)
Zhibin	Lu			Princess Margaret Cancer Centre	Toronto, Ontario, Canada		
Trevor	Pugh			Princess Margaret Cancer Centre	Toronto, Ontario, Canada		
Lillian	Siu			Princess Margaret Cancer Centre	Toronto, Ontario, Canada		
Stuart	Watt			Princess Margaret Cancer Centre	Toronto, Ontario, Canada		
Natasha	Leighl			Princess Margaret Cancer Centre	Toronto, Ontario, Canada		
Celeste	Yu			Princess Margaret Cancer Centre	Toronto, Ontario, Canada		
Lailah	Ahmed			Princess Margaret Cancer Centre	Toronto, Ontario, Canada		
Geeta	Krishna			Princess Margaret Cancer Centre	Toronto, Ontario, Canada		
Carlos	Virtaenen			Princess Margaret Cancer Centre	Toronto, Ontario, Canada		
Helen	Chow			Princess Margaret Cancer Centre	Toronto, Ontario, Canada		
Demi	Plagianakos			Princess Margaret Cancer Centre	Toronto, Ontario, Canada		
Samantha	Del Rossi			Princess Margaret Cancer Centre	Toronto, Ontario, Canada		
Nitthusha	Singaravelan			Princess Margaret Cancer Centre	Toronto, Ontario, Canada		
Sevan	Hakgor			Princess Margaret Cancer Centre	Toronto, Ontario, Canada		
Nazish	Qazi			Princess Margaret Cancer Centre	Toronto, Ontario, Canada		

© 2020 American Medical Association. All rights reserved.

Supplemental Online Content: Nonauthor Collaborators

*Indicates required information. Only first name, last name, and suffix will appear in PubMed.

***First Name and Middle Initial(s)**	***Last Name**	***Suffix (eg, Jr, III)**	Academic Degrees	Institution	Location (city, state/province, country)	Role or Contribution, eg, chair, principal investigator	Group (if more than 1 Group listed in the byline) and/or Subgroup (eg, Steering Committee)
Alisha	Nguyen			Princess Margaret Cancer Centre	Toronto, Ontario, Canada		
Natalie	Stickle			Princess Margaret Cancer Centre	Toronto, Ontario, Canada		
Thomas	Stricker			Vanderbilt-Ingram Cancer Center	Nashville, TN		
Christine	Micheel			Vanderbilt-Ingram Cancer Center	Nashville, TN		
Ingrid	Anderson			Vanderbilt-Ingram Cancer Center	Nashville, TN		
Leigh	Jones			Vanderbilt-Ingram Cancer Center	Nashville, TN		
Lucy	Wang			Vanderbilt-Ingram Cancer Center	Nashville, TN		
Christine	Lovly			Vanderbilt-Ingram Cancer Center	Nashville, TN		
Lucy	Wang			Vanderbilt-Ingram Cancer Center	Nashville, TN		
Michele	LeNoue Newton			Vanderbilt-Ingram Cancer Center	Nashville, TN		
Ben	Park			Vanderbilt-Ingram Cancer Center	Nashville, TN		
Jeremy	Warner			Vanderbilt-Ingram Cancer Center	Nashville, TN		
Daniel	Fabbri			Vanderbilt-Ingram Cancer Center	Nashville, TN		
Joseph	Coco			Vanderbilt-Ingram Cancer Center	Nashville, TN		
Chen	Ye			Vanderbilt-Ingram Cancer Center	Nashville, TN		

© 2020 American Medical Association. All rights reserved.

Supplemental Online Content: Nonauthor Collaborators

*Indicates required information. Only first name, last name, and suffix will appear in PubMed.

***First Name and Middle Initial(s)**	***Last Name**	***Suffix (eg, Jr, III)**	Academic Degrees	Institution	Location (city, state/province, country)	Role or Contribution, eg, chair, principal investigator	Group (if more than 1 Group listed in the byline) and/or Subgroup (eg, Steering Committee)
Sandip	Chaugai			Vanderbilt-Ingram Cancer Center	Nashville, TN		
Sanjay	Mishra			Vanderbilt-Ingram Cancer Center	Nashville, TN		
Yuanchu James	Yang			Vanderbilt-Ingram Cancer Center	Nashville, TN		
Li	Wen			Vanderbilt-Ingram Cancer Center	Nashville, TN		
Rodrigo	Dienstmann			Vall d’ Hebron Institute of Oncology	Barcelona, Spain		
Susana	Aguilar Izquierdo			Vall d’ Hebron Institute of Oncology	Barcelona, Spain		
Cristina	Viaplana Donato			Vall d’ Hebron Institute of Oncology	Barcelona, Spain		
Francesco	Mancuso			Vall d’ Hebron Institute of Oncology	Barcelona, Spain		
Umit	Topaloglu			Wake Forest University Health Sciences (Wake Forest Baptist Medical Center)	Winston-Salem, NC		
Liang	Liu			Wake Forest University Health Sciences (Wake Forest Baptist Medical Center)	Winston-Salem, NC		
Meijian	Guan			Wake Forest University Health Sciences (Wake Forest Baptist Medical Center)	Winston-Salem, NC		
Wei	Zhang			Wake Forest University Health Sciences (Wake Forest Baptist Medical Center)	Winston-Salem, NC		

© 2020 American Medical Association. All rights reserved.

Supplemental Online Content: Nonauthor Collaborators

*Indicates required information. Only first name, last name, and suffix will appear in PubMed.

***First Name and Middle Initial(s)**	***Last Name**	***Suffix (eg, Jr, III)**	Academic Degrees	Institution	Location (city, state/province, country)	Role or Contribution, eg, chair, principal investigator	Group (if more than 1 Group listed in the byline) and/or Subgroup (eg, Steering Committee)
Guangxu	Jin			Wake Forest University Health Sciences (Wake Forest Baptist Medical Center)	Winston-Salem, NC		
James	Knight			Yale University Cancer Center	New Haven, CT		
Michael	D'Eletto			Yale University Cancer Center	New Haven, CT		
E. Zeynep	Ormay			Yale University Cancer Center	New Haven, CT		
Shrikant	Mane			Yale University Cancer Center	New Haven, CT		
Kaya	Bilguvar			Yale University Cancer Center	New Haven, CT		
Walther	Zenta			Yale University Cancer Center	New Haven, CT		
Daniel	Dykas			Yale University Cancer Center	New Haven, CT		

© 2020 American Medical Association. All rights reserved.
